# Human Papillomavirus Vaccination and Social Media: Results in a Trial With Mothers of Daughters Aged 14–17

**DOI:** 10.3389/fdgth.2021.683034

**Published:** 2021-09-03

**Authors:** David B. Buller, Sherry Pagoto, Kimberly Henry, Julia Berteletti, Barbara J. Walkosz, Jessica Bibeau, Katie Baker, Joel Hillhouse, Kelsey M. Arroyo

**Affiliations:** ^1^Klein Buendel, Inc., Golden, CO, United States; ^2^Department of Allied Health Sciences, University of Connecticut, Storrs, CT, United States; ^3^Department of Psychology, Colorado State University, Fort Collins, CO, United States; ^4^Department of Community and Behavioral Health, East Tennessee State University, Johnson City, TN, United States; ^5^Department of Clinical and Health Psychology, University of Florida, Gainesville, FL, United States

**Keywords:** human papillomavirus, vaccine, social media, mothers, adolescents

## Abstract

**Introduction:** Parents acquire information about human papillomavirus (HPV) vaccines online and encounter vaccine-critical content, especially on social media, which may depress vaccine uptake. Secondary analysis in a randomized trial of a Facebook-delivered adolescent health campaign targeting mothers with posts on HPV vaccination was undertaken with the aims of (a) determining whether the pre–post-change occurred in self-reports of the mothers on HPV vaccination of their adolescent daughters; (b) describing the comments and reactions to vaccine posts; (c) exploring the relationship of campaign engagement of the mothers assessed by their comments and reactions to posts to change in the self-reports of the mothers of HPV vaccination.

**Materials and Methods:** Mothers of daughters aged 14–17 were recruited from 34 states of the US (*n* = 869). A social media campaign was delivered in two Facebook private groups that differed in that 16% of posts in one were focused on indoor tanning (IT) and 16% in the other, on prescription drug misuse, assigned by randomization. In both groups, posts promoted HPV vaccination (*n* = 38 posts; no randomization) and vaccination for other disease (e.g., influenza, *n* = 49). HPV and other vaccination posts covered the need for a vaccine, the number of adolescents vaccinated, how vaccines are decreasing the infection rates, and stories of positive benefits of being vaccinated or harms from not vaccinating. Guided by social cognitive theory and diffusion of innovations theory, posts were intended to increase knowledge, perceived risk, response efficacy (i.e., a relative advantage over not vaccinated daughters), and norms for vaccination. Some vaccination posts linked to stories to capitalize on identification effects in narratives, as explained in transportation theory. All mothers received the posts on vaccination (i.e., there was no randomization). Mothers completed surveys at baseline and 12- and 18-month follow-up to assess HPV vaccine uptake by self-report measures. Reactions (such as sad, angry) and comments to each HPV-related post were counted and coded.

**Results:** Initiation of HPV vaccination (1 dose) was reported by 63.4% of mothers at baseline, 71.3% at 12-month posttest (pre/post *p* < 0.001), and 73.3% at 18-month posttest (pre/post *p* < 0.001). Completion of HPV vaccination (two or three doses) was conveyed by 50.2% of mothers at baseline, 62.5% at 12-month posttest (pre/post *p* < 0.001), and 65.9% at 18-month posttest (pre/post *p* < 0.001). For posts on HPV vaccines, 8.1% of mothers reacted (*n* = 162 total), and 68.4% of posts received a reaction (63.2% like; 13.2% love, 7.9% sad). In addition, 7.6% of mothers commented (*n* = 122; 51 unfavorable, 68 favorable, 1 neutral), and 50.0% of these posts received a comment. There were no differences in pre–post change in vaccine status by the count of reactions or comments to HPV vaccine posts (Ps > 0.05). Baseline vaccination was associated with the valence of comments to HPV vaccine posts (7.2% of mothers whose daughters had completed the HPV series at baseline made a favorable comment but 7.6% of mothers whose daughters were unvaccinated made an unfavorable comment).

**Conclusion:** Effective strategies are needed in social media to promote HPV vaccines and counter misinformation about and resistance to them. Mothers whose daughters complete the HPV vaccine course might be recruited as influencers on HPV vaccines, as they may be predisposed to talk favorably about the vaccine. Comments from mothers who have not been vaccinated should be monitored to ensure that they do not spread vaccine-critical misinformation. Study limitations included lack of randomization and control group, relatively small number of messages on HPV vaccines, long measurement intervals, inability to measure views of vaccination posts, reduced generalizability related to ethnicity and social media use, and use of self-reported vaccine status.

**Clinical Trial Registration:** www.clinicaltrials.gov, identifier NCT02835807.

## Introduction

Despite the recommendation of the Advisory Committee on Immunization Practices (ACIP), (1) only 54% of adolescents aged 13–17 were up-to-date for the human papillomavirus (HPV) vaccine in 2019 (women 57%; men: 52%) (2), far below the healthy people 2030 target of 80%. Vaccine initiation and completion are affected by health beliefs (e.g., vaccine knowledge; the importance of preventive vaccinations; side effects concerns) (3–6) which are amenable to change through health education interventions. Identifying effective strategies to improve HPV vaccination rates is a national and international priority (7, 8).

Parents frequently use the Internet as a reliable source of information on child health (9–11), and online interventions may reach large proportions of parents whose children are not up-to-date on the HPV vaccine. Parents acquire information about HPV vaccines online (12–15), but unfortunately, inaccurate, misleading, unsupported, and harmful information can be circulated online (16, 17), including about vaccines (12, 18–22). False claims are made that HPV vaccines increase teen sexual activity; a low prevalence of HPV-related diseases exists; other modes of prevention are available; HPV vaccines are unsafe due to insufficient testing; HPV vaccines have severe side effects or cause death; HPV vaccine regulations are a product of corruption or conspiracies; and HPV vaccines violate civil liberties (15, 22–27). HPV vaccination decisions of the parents are affected by this online content (12, 28–31) which can depress vaccine uptake (12, 28–31). For instance, in one survey, parents who heard stories about only harms (e.g., mild side effects and death) were unlikely to vaccinate children for HPV even if they also heard stories about disease prevention (12).

Social media, in particular, spread information on HPV vaccines and transmit vaccine-critical content (12, 32–34). Growing research on social media, particularly related to vaccines, finds widespread misinformation and unsubstantiated claims about corruption, conspiracies, and distrust in vaccine regulations, especially of drug companies, government agencies, and physicians (35). Parents have reported receiving stories about the harms of HPV vaccines on social media and news media, while stories on the prevention of HPV disease occurred in conversations (12). In one study, mothers who first learned about HPV vaccines through social media tended not to vaccinate daughters, while those first hearing about it from their general practitioner vaccinated them (36). There have been calls for efforts to improve accurate HPV content on social media and correct misinformation (37–41) to improve clinical encounters on HPV vaccines (32).

Much of the past research on HPV vaccine content in social media has been descriptive and correlational (21, 26, 42), with few studies examining the impact of HPV vaccine messages prospectively (43, 44). The authors recently completed a randomized trial on a 12-month social media adolescent health campaign delivered to mothers of daughters aged 14–17, which contained posts promoting HPV vaccination. A unique feature of social media is the presence of user-generated content (e.g., comments and reactions), and an analysis of the first 10 posts on HPV vaccination in the campaign (45) revealed that about 10% of mothers reacted/commented on HPV vaccine posts. Mothers posting supportive comments were more likely to have vaccinated daughters at baseline, as had those remaining silent, while mothers posting critical comments were less likely to have vaccinated daughters. In this study, we present analyses of pre-post change in reports of the mothers on vaccination of their adolescent daughters after the entire year-long social media campaign, a description of comments and reactions to all vaccine posts, and the relationship of campaign engagement of the mothers to comments and reactions.

## Materials and Methods

### Trial Design

Mothers with teenage daughters were enrolled in a randomized controlled trial with the primary purpose of evaluating the effect of social media posts on their permissiveness toward indoor tanning (IT) by daughters. After a baseline survey, mothers were randomly assigned to one of two Facebook private groups. Randomization was accomplished by the project biostatistician, using permuted-block randomization (block size = 2). All mothers received a Facebook feed of posts on adolescent health topics, mother–daughter communication and current events, which differed on whether the feed included posts on IT (intervention) or prescription drug misuse (control). Both feeds contained posts on HPV vaccination and vaccination for other diseases (e.g., influenza) as there was no randomization on the presence of the HPV vaccination messages. A community manager added mothers to the groups, scheduled posts, and monitored and responded to comments during the 12-month campaign, after which mothers completed posttest surveys at 12- and 18-months post-randomization. To retain participants, any mothers who left the private groups were contacted and asked to re-join and mothers were alerted to upcoming posttests and compensated ($40 for baseline, $20 for 12-month posttest, and $40 for 18-month posttest). Daughters were invited to complete the baseline survey and both posttests (compensation = $20, $15, and $25, respectively) but were not enrolled in the social media feed. Study staff other than the community manager and project coordinator were blinded. The Western Institutional Review Board (IRB) and the IRBs at East Tennessee State University and the University of Connecticut approved study protocols.

### Participants

Between May 2017 and June 2018, mothers were enrolled who met inclusion criteria: (1) having a daughter aged 14–17; (2) living in one of 34 U.S. states without a complete ban on IT by minors; (3) reading English; (4) having a Facebook account and logging in at least once per week; and (5) willing to “friend” the community manager of the project to join a private Facebook group. Mothers were excluded if they were unable to read English or did not consent and “friend” the community manager. Initially, mothers were recruited in Tennessee through community-based methods (e.g., working with Coordinated School Health coordinators, presentations at community events, and outcalls from a survey center). When these methods did not yield a sufficient number of mothers, Qualtrics was contracted and recruited mothers from its survey panel in 33 other states. All mothers were blind to treatment because they received a social media feed whose purpose was described as providing information on adolescent health and mother-daughter communication. Statistical power calculations provided a target sample size of 860 that would achieve 80% power for small to moderate effects. Mothers provided email addresses for daughters and parental consent, and daughters were invited to complete assessments, providing informed assent. To avoid a major recruitment barrier, the participation of the daughter was not required. When mothers had more than one daughter, the one with the nearest birthday was selected.

### Intervention

The research team developed a social media intervention, named *Health Chat*, using principles of social cognitive theory (SCT) (46), transportation theory (TT) (47), and diffusion of innovations theory (DIT) (48). The campaign also covered skills for communicating with teens (i.e., active listening, self-disclosure, empathy, and conflict management). Posts sought to create transportation into and identification with stories by linking to narratives from mothers and daughters about health risks, not giving permission for risky behaviors, and avoiding engaging in risky behaviors oneself (47, 49). Posts referenced current events and public figures to heighten the engagement of the mothers and encouraged mothers to react to (e.g., like) and comment on posts to evoke social comparison processes that can build norms (50, 51). Posts included social norms-based appeals, appearance-based messaging, and health-risk messaging.

Messages were created by investigators and reviewed by the entire team for acceptability and readability. Initial messages were pretested in a pilot feed with mothers (*n* = 90) not in the trial and changes were made to enhance aesthetics, message clarity, and engagement based on the results. Messages addressing current events were created during the intervention. Approximately 84% of posts addressed adolescent health topics and mother–daughter communication. Topics included vaccinations (e.g., influenza and human papillomavirus), mental health (e.g., stress and bullying), substance use (e.g., alcohol, cannabis, and tobacco), healthy lifestyles (e.g., physical activity and nutrition), media literacy, and general parenting (e.g., college preparation). They were selected based on formative research with mothers, engagement of the mothers during pilot testing, or emerging issues in comments of the mothers during the campaign. About 16% of posts focused on preventing IT (intervention group) or prescription drug misuse (control group). The two private Facebook groups received the same feed of posts except for the manipulated posts on IT or prescription drug misuse.

Several techniques were used in an attempt to increase engagement by mothers with the social media feed. Almost all posts included an image or infographic, along with the text. Some included links to outside sources. Many posts included a question or conversation starter, such as “Do you know if your daughters is up-to-date on her vaccines?” to invite mothers to react to and comment on posts. Finally, posts on topics of high human interest but not tied to any of the specific health topics were included periodically in the feed, such as soliciting favorite recipes or book recommendations.

A group of posts promoted vaccination for HPV (*n* = 38 posts) and general vaccine information, including influenza vaccination (n = 49 posts). Regarding HPV, posts covered the need for the vaccine (perceived risk), percent of adolescents vaccinated, the proportion of parents choosing to vaccinate children against HPV (descriptive norms), how HPV vaccines are decreasing infection rates (response efficacy), and stories of women who died from cervical cancer (perceived severity) or parents who decided to vaccinate their children (identification). Posts on vaccination for other diseases addressed misinformation surrounding vaccines, the need for annual influenza shots (risk), vaccine safety and efficacy (response efficacy), adolescent vaccine schedules (how-to knowledge), and reducing barriers to vaccination. Posts were in didactic (e.g., providing facts about rates of HPV) and narrative (e.g., sharing a story about someone who died from cervical cancer) format. Narratives were intended to influence through a process of identification with the characters in the stories. The primary focus on communicating with mothers was appropriate as they drive decisions about HPV vaccines (52–54) although HPV vaccination may be one of the first opportunities to engage adolescent daughters in healthcare decision-making (55). The posts on HPV and other vaccinations were not randomly assigned; the feeds in both Facebook groups included these posts.

The social media campaign was run in two private Facebook groups. In these groups, posts, comments, reactions, and membership were only viewable to participants and they could not share group content with Facebook users outside the group. This prevented contamination. Messages were posted two times a day to each group over 12 months (~710 total posts). This rate was designed to be sufficient to influence but avoid message fatigue. A community manager scheduled posts, monitored reactions/comments, and replied to misinformation. In addition, mothers received a bi-weekly email newsletter highlighting the most popular recent posts.

### Measures

#### Primary Outcomes

The primary outcome measures in the baseline and 12- and 18-month posttest surveys in this analysis were self-reports of HPV vaccination by mothers. Mothers were asked if the daughter had been vaccinated for HPV and if so, how many shots had she received. Initiation of vaccination was defined as receipt of one-shot of HPV vaccine and completion was defined as two or three shots (two shots are recommended for girls under age 15 while three shots are recommended for girls aged 15–17). Daughters were asked these same questions for themselves.

#### Demographics

Demographic characteristics were collected, namely, mother and daughter age and skin phenotype (e.g., eye color and hair color) (56). Mothers also reported on personal and family history of skin cancer and political ideology.

#### Engagement

The engagement of the mothers with the HPV vaccination posts was measured by counting the number of reactions (i.e., like, love, and sad buttons) and comments to each post. Comments and reactions to posts on HPV and other vaccines were extracted by a trained research assistant at the end of the social media campaign, using Grytics software. The content of reactions was recorded (i.e., like, angry, love, haha, wow). Further, the content in the comments was coded by trained research staff. The comments were coded as favorable (i.e., positive discussion of HPV vaccine or statement daughter was vaccinated), unfavorable (i.e., critical of HPV vaccine or statement of hesitancy or refusal to vaccinate daughter), or neutral (i.e., part of general group dialogue but not related to HPV vaccination specifically). Each comment was coded by one research assistant and 60% were coded by a second coder to check inter-rater reliability (Krippendorff's alpha = 0.76). Emergent themes were then identified based on content codes.

### Statistical Analysis

Descriptive statistics were computed using SAS, Version 9.3. *F*-tests were utilized to determine if increases in HPV vaccination rates were statistically significant. Correlation coefficients were computed to compare mother and daughter reports. Multinomial logistic regression was fit to identify predictors of vaccine uptake from pre- to post-intervention. Alpha criterion level of 0.05 was set for all tests.

## Results

### Profile of Sample

Demographic characteristics of the 869 mothers and 469 daughters enrolled in the study have been reported elsewhere (57). Briefly, mothers had a mean age of 43.1 years (SD = 6.6) and were 82.4% non-Hispanic white, 57.8% had a college education, and 51.1% had household incomes over $80,000. They were diverse on political ideology, with 24.5% conservative and 23.8% liberal, with the remaining half (51.7%), middle of the road. The daughters had a mean age of 15.3 years, 74.7% were non-Hispanic whites, and 24.8% had a high-risk skin type.

### HPV Vaccination Rates

At baseline, 63.4% of mothers reported that their daughters had received at least one dose of the HPV vaccine, with 50.2% saying they had received two or three doses (i.e., possibly completed series, depending on the age of the daughter). At the 12-month posttest, 71.3% of mothers reported that daughters had received at least one dose of the HPV vaccine (pre/post comparison *F* = 14.05, *p* < 0.001) and 62.5% reported that daughters had received two or three vaccine doses (pre/post comparison *F* = 21.31, *p* < 0.001). Looking just at mothers whose daughters had not completed the HPV vaccine series at baseline (*n* = 293, 227 with no shots and 66 with one shot), 18.5% of daughters with no shots at baseline and 53.0% of those who had received one shot at baseline had completed the series at the 12-month follow-up (χ^2^ = 31.46, *p* < 0.001). At the 18-month posttest, 73.3% of mothers reported that daughters had received at least one dose of the HPV vaccine (pre/post comparison *F* = 20.15, *p* < 0.001), and 65.9% reported that daughters had received two or three vaccine doses (pre/post comparison *F* = 38.05, *p* < 0.001). Again, subsetting mothers whose daughters had not completed the HPV vaccine series at pretest (*n* = 328, 248 with no shots and 80 with one-shot), 22.2% of daughters with no shots at baseline, and 70.0% of those who had received one shot at baseline had completed the series at the 18-month follow-up (χ^2^ = 61.79, *p* < 0.001). Reports of the mothers of HPV vaccine uptake were corroborated by daughters (82.1–88.4% correspondence, *r* = 0.65–0.76, *p* < 0.001).

### Content and Valence of Reactions and Comments to HPV Posts

For social media posts on HPV vaccines (*n* = 38), 8.1% of mothers reacted to a post (*n* = 162 reactions total), and 68.4% of all HPV vaccine posts received a reaction (63.2% like; 13.2% love, 7.9% sad). In addition, 7.6% of mothers commented on an HPV vaccine post (*n* = 123 comments total; 54 unfavorable, 68 favorable, 1 neutral), and 50.0% of all HPV vaccine posts received a comment. Similarly, for posts on other vaccines, 5.4% of mothers reacted to a post (*n* = 97 reactions total) and 71.4% of the posts on other vaccines received a reaction (59.2% like; 0.0% love; 12.2% sad). In addition, 4.6% of mothers commented on posts on other vaccines (*n* = 67 comments total; 14 unfavorable, 48 favorable, 5 neutral), and 42.9% of these posts on other vaccines received a comment. Looking at all posts in the feed, 55.8% of mothers reacted to a post and 68.2% of posts received a reaction. In addition, 58.5% of mothers commented on a post and 53.8% of posts received a comment.

Content analysis of all vaccination comments was done to explore themes for both favorable and unfavorable comments (Table 1). Favorable themes included the daughters were vaccinated, boys, as well as girls, should be vaccinated, benefits of vaccines outweigh the risks, vaccines reduced rates of disease, and physician supported vaccination. Unfavorable themes included daughter received certain vaccines but not others; lack of efficacy, safety concerns, or fear of unknown long-term side effects; negative stories or vague unfavorable “issues” regarding vaccination; mistrust in sources promoting vaccines; and lack of physician support for vaccination. In addition, 4.9% of comments on HPV posts mentioned sexual activity related to decision-making about getting daughters vaccinated.

**Table 1:** Themes in the comments to posts on human papillomavirus (HPV) and other vaccinations.

**Themes**	**HPV vaccine posts**		**Other vaccine posts**	
	***N***	**%**	***N***	**%**
**Favorable comments**				
The daughter was vaccinated	42	34.1	33	49.3
Boys as well as girls should be vaccinated	12	9.8	0	0.0
Benefits of vaccines outweigh the risks	7	5.7	5	7.5
Vaccines reduce the rate of disease	5	4.1	3	4.5
Physician supports vaccination	2	1.6	1	1.5
**Unfavorable comments**				
Daughter received certain vaccines but not other, or their children received different vaccines from one another	18	14.6	3	4.5
Lack of efficacy, safety concerns, or fear of unknown long-term side effects	14	11.4	9	13.4
Negative stories or vague unfavorable issues regarding vaccination	11	8.9	1	1.5
Mistrust in organizations promoting vaccines	3	2.4	6	9.0
Lack of physician support for vaccination	8	6.5	0	0.0

### Engagement With HPV Posts

We explored the relationship between the engagement of the mothers with HPV and other vaccine posts and the status of the vaccine of their daughters. Specifically, we explored whether reactions and comments were associated with pre/post-change in vaccine status. We fit three multinomial logistic regressions, subsetting to include only mothers who reported that their daughters had not completed the HPV vaccine series at baseline. Counts of reactions and comments to HPV vaccine posts (zero vs. 2/3 doses: estimate = −0.24, *t* = 0.59, *p* = 0.56; 1 vs. 2/3 doses: estimate = −0.26, *t* = −0.37, *p* = 0.71) and other vaccine posts (estimate = −117, *t* = −1.08, *p* = 0.28; estimate = 0.04, *t* = 0.08, *p* = 0.93) did not differ by vaccine status at 18-month posttest.

Instead, baseline vaccine status was associated with the valence of comments to HPV vaccine posts (Table 2). More vaccine-favorable comments to HPV vaccine posts were made by mothers whose daughters had completed the HPV series at baseline. A few mothers whose daughters were unvaccinated also made favorable comments but mothers whose daughters had initiated but not completed the series made very few favorable comments. By contrast, vaccine-unfavorable comments were made primarily by mothers whose daughters were not vaccinated at baseline. Mothers whose daughters had completed the HPV series at baseline or had initiated but not completed the series made almost no unfavorable comments to the HPV vaccination posts. Although mothers made fewer comments to posts on other vaccines, this same pattern emerged among mothers based on the status of the baseline vaccination of the daughters.

**Table 2:** Valence of comments to HPV and other vaccination posts by HPV vaccine status of daughter at baseline.

**Valence of comment**	**Baseline HPV vaccination status**		
	**Not vaccinated**	**1 dose**	2/3 doses^*^
N	317	104	405
**HPV vaccination posts**
Favorable	3.5%	1.0%	7.2%
Unfavorable	7.6%	0.0%	1.2%
**Other vaccination posts**
Favorable	3.2%	1.0%	3.7%
Unfavorable	2.5%	0.0%	0.5%

* *Assumed to have completed HPV vaccination series*.

## Discussion

Human papillomavirus vaccine uptake for daughters increased during the 12-month period of the social media campaign. The largest increase appeared in the completion of the series, with smaller changes in the initiation of the series. It is not clear in the pre–post comparison whether these changes were due to the social media campaign, especially since engagement with posts was not related to the pre–post increase in HPV vaccine uptake. Unfortunately, Facebook stopped reporting whether participants viewed posts during the study, so we were limited to counting comments and reactions as indicators of engagement. However, mothers could have viewed posts without commenting on or reacting to them. Other research has shown that users following social media pages who view but do not comment may still be engaged with the content but may be concerned about privacy or have stronger information needs than social interaction needs (58–61). These users can be affected by the posts and pass the information along to others (61, 62). Thus, the number of comments and reactions may have under-estimated actual engagement with the posts in this study.

The effect of the campaign on the completion of the HPV vaccine series may have been slightly greater than on initiation of it for daughters. These mothers may be more inclined to vaccinate daughters than those who had not initiated the series in general, although some remained incomplete at the posttests. The campaign may have nudged some mothers who had started the HPV vaccine series for their daughters prior to the study to take steps to complete the series during the campaign or in the 6 months after it ended, as 70% of mothers whose daughters had received only one shot at baseline reported they had completed the series by the 18-month posttest. A much smaller proportion (18% at 12 months and 22% at 18 months) of those who had not yet vaccinated their daughters at the beginning of the trial had completed the HPV vaccine series at either posttest than those whose daughters who had already had one dose at baseline (53% and 70%).

A sizable group of mothers (27%) had daughters who remained unvaccinated throughout the trial and this group was more likely to post unfavorable comments, suggesting that some of these mothers with unvaccinated daughters were actively resistant to HPV posts. Vaccine-critical comments might be used to identify mothers who are resistant to HPV vaccines and tailor posts to respond to reasons for vaccine hesitancy expressed in their comments. By contrast, mothers who had initiated but not completed HPV vaccination did not comment much either favorably or unfavorably on posts about it. This latter group may have been uncertain about whether to complete it or had barriers to completion, but they were also not strongly resistant to the vaccine. The social media campaign may have nudged some of them to get the HPV vaccine series completed.

Less than 10% of mothers engaged with the posts on HPV vaccination (i.e., reacted or commented) but the number of posts that received a reaction or comment was similar in rate to all posts in the feed. The most common comment in response to HPV vaccine posts was a mother sharing that she had her daughter vaccinated. For this reason, mothers who complete the HPV vaccine series for their daughters might be recruited as influencers on HPV vaccines in future vaccine-promotion programs, as some appear predisposed to talk favorably about the vaccine. However, they may need to be instructed on how to make comments that are likely to influence other mothers because many just simply commented that they had vaccinated their daughters without providing other information that might be influential such as noting the benefits of disease prevention or that physicians recommended it. This same tendency, seen in an early analysis of comments to initial posts (45), continued throughout the social media campaign. Simple statements may help increase perceived descriptive norms for HPV vaccination, which might influence some mothers to vaccinate their daughters (63, 64). But, for many hesitant or resistant mothers, simply providing more information or fact-checking misinformation may not be sufficient. Additional strategies, such as counter-narratives, peer correction, factual elaboration, coherence/credibility appeals, and developing media and e-Health literacy skills (45, 65–71), may be needed to dispel the concerns about lack of efficacy, safety and harmful side-effects, and mistrust of organizations and agencies promoting HPV vaccines. These same vaccine-critical comments about safety and efficacy have been documented in other studies of social media content on HPV vaccines (72–79) and seem to have resonated with some mothers. Sharing stories about why mothers vaccinated daughters for HPV may help correct misinformation and overcome mistrust as stories can influence through identifying with characters, shifting social norms, and reducing counter-arguments by being transported into the stories (49, 80–82). Some mothers shared stories in unfavorable comments in this study and others (22, 23), and they may have had a strong negative impact. Comments and reactions from mothers who have not vaccinated daughters for HPV should also be monitored for misinformation on vaccines, which should be addressed quickly to help forestall it from going viral (65).

The comments that physicians did not support HPV vaccination need to be countered as medical professionals can be important, credible, and influential sources of information on HPV vaccination (14, 83–85). Mistrust in organizations and agencies promoting the HPV vaccine has been observed in other analyses (72, 74, 76, 86) and may be instrumental for hesitant or resistant mothers. Derogating the source is one way to reduce the perceived risk for not vaccinating their daughters when they have decided not to use it and rely instead on less effective prevention methods (87, 88).

### Limitations

The study had several limitations. The lack of randomization and absence of a control group undermined conclusions about whether the social media campaign produced the observed change in HPV vaccination rates at posttest. The number of messages specifically on HPV vaccination was small (about 5% of all posts) but they were supplemented by nearly 50 posts on other vaccinations (e.g., influenza) which should have improved vaccination intentions in general. The measurement times at 12- and 18-months post-randomization were long and risk history effects, where secular events (changes in personal health history; visits to physician offices, and other media coverage on HPV, vaccines, or health topics) could have occurred after the HPV vaccination messages that influenced decisions of the parents to vaccinate daughters. Once again, the lack of randomization meant the design could not control for this threat to internal validity. The inability to collect data on views from the Facebook feed meant we could not determine whether mothers saw the HPV and other vaccination posts. The decision by Facebook to eliminate reports on views of posts in the private groups was out of our control and occurred after the study was launched. We contacted Facebook and requested access to the views data but they were unable to provide it. Instead, we decided to use counts of reactions and comments to the vaccination posts, which were still reported by Facebook, as indicators of viewership, but as noted above these likely under-estimated actual engagement with the vaccination posts. It is worth noting that the lack of an objective measure of exposure to campaign messages by individual respondents often is limited in community-based evaluations of public health campaigns that rely on other media such as television, radio, print, and billboards. While the sample was large and included mothers from 34 states of the US, it may have limited generalizability because mothers were predominately non-Hispanic white and had regular social media use, the Qualtrics survey panel tends toward participants with higher socioeconomic status, mothers who chose to participate were interested in their health of the daughters, mothers lived in states that may be less socially progressive as they did not have bans on indoor tanning by minors, and daughters were older than the first age at which HPV vaccination is recommended. Self-reports of vaccine status of the mothers also may be biased but these measures have shown good specificity and sensitivity in past surveys (89–91) and produced estimates similar to government immunization records (92). Further, they were corroborated by reports of the daughters. The limitations were offset somewhat by strengths in the study: mothers were enrolled and pretested prior to the social media campaign and a pre-post change was observed, rather than inferring pre-existing vaccination beliefs and actions of the mothers.

#### Conclusion

Human papillomavirus vaccination rates in the United States continue to lag national health goals. Social media is a major source of health information and supportive and critical information on HPV vaccination. Effective strategies are needed in social media to promote HPV vaccines and counter misinformation about them to move mothers who are resisting vaccination. Interspersing vaccine messages in a feed for parents may be effective, as it can expose them to a large number of messages. Even if social media messaging does not change the minds of highly resistant mothers who contribute unfavorable comments, carefully crafted messages may convince mothers who are uninformed, uncertain, or not currently taking action to vaccine their daughters to ignore or resist the false claims and misinformation about the HPV vaccine and complete the series for their daughters.

## Data Availability Statement

The original contributions presented in the study are included in the article/supplementary material, further inquiries can be directed to the corresponding author/s.

## Ethics Statement

The studies involving human participants were reviewed and approved by The Western Institutional Review Board and the IRBs at East Tennessee State University and University of Connecticut. Written informed consent to participate in this study was provided by the participants' legal guardian/next of kin.

## Author Contributions

DB: conceptualization, methodology, verification, investigation, writing—original draft, visualization, supervision, project administration, and funding acquisition. SP: conceptualization, methodology, verification, investigation, resources, writing—review and editing, supervision, project administration, and funding acquisition. KH: conceptualization, methodology, formal analysis, writing—review and editing, and funding acquisition. JBe: verification, validation, investigation, resources, data curation, writing—review and editing, and supervision. BW: conceptualization, methodology, investigation, writing—review and editing, resources, and funding acquisition. JBi: verification, validation, investigation, resources, data curation, writing—review and editing, and supervision. KB: conceptualization, methodology, investigation, writing—review and editing, supervision, project administration, and funding acquisition. JH: conceptualization, methodology, investigation, writing—review and editing, and funding acquisition. KA: verification, validation, investigation, data curation, and writing—review and editing. All authors contributed to the article and approved the submitted version.

## Conflict of Interest

DB's spouse is an owner of Klein Buendel, Inc. and he receives a salary from Klein Buendel. JBe and BW receive a salary from Klein Buendel, Inc. The remaining authors declare that the research was conducted in the absence of any commercial or financial relationships that could be construed as a potential conflict of interest.

## Publisher's Note

All claims expressed in this article are solely those of the authors and do not necessarily represent those of their affiliated organizations, or those of the publisher, the editors and the reviewers. Any product that may be evaluated in this article, or claim that may be made by its manufacturer, is not guaranteed or endorsed by the publisher.
